# Unlocking the Fatty Acid and Antioxidant Profile of Grape Pomace: A Systematic Assessment Across Varieties and Vintages for Its Sustainable Valorization

**DOI:** 10.3390/molecules30153150

**Published:** 2025-07-28

**Authors:** Teresa Abreu, Rui Ferreira, Paula C. Castilho, José S. Câmara, Juan Teixeira, Rosa Perestrelo

**Affiliations:** 1 CQM—Centro de Química da Madeira, Universidade da Madeira, Campus da Penteada, 9020-105 Funchal, Portugal; teresa.abreu@staff.uma.pt (T.A.); rui.ferreira@staff.uma.pt (R.F.); pcastilho@staff.uma.pt (P.C.C.); jsc@staff.uma.pt (J.S.C.); 2 Departamento de Química, Faculdade de Ciências Exatas e Engenharia, Universidade da Madeira, Campus da Penteada, 9020-105 Funchal, Portugal; 3 Parque Industrial da Cancela, Justino´s Madeira Wines, S.A., Caniço, 9125-042 Santa Cruz, Portugal; juan.teixeira@justinosmadeira.com

**Keywords:** grape pomace, fatty acid profile, antioxidant activity, circular economy, nutraceuticals

## Abstract

Grape pomace (GP), the main by-product of the wine industry, represents a valuable source of bioactive metabolites with significant potential for valorization in the context of sustainable bioresource management. This study systematically characterizes the fatty acid methyl ester (FAME) profile, total phenolic content (TPC), total flavonoid content (TFC), and antioxidant activities (DPPH, ABTS, ORAC) of GP derived from seven grape varieties across three consecutive vintages (2022–2024). White GP, particularly Verdelho and Sercial, exhibited a superior lipid quality with high concentrations of methyl linoleate (up to 1997 mg/100 g DW) and methyl oleate (up to 1294 mg/100 g DW), low atherogenic (AI < 0.05) and thrombogenic indices (TI ≤ 0.13), and elevated PUFA/SFA ratios (≥8.2). In contrast, red GP, especially from Complexa and Tinta Negra, demonstrated the highest antioxidant potential, with TPC values up to 6687 mgGAE/100 g DW, TFC up to 4624 mgQE/100 g DW, and antioxidant activities reaching 5399 mgTE/100 g (DPPH) and 7219 mgTE/100 g (ABTS). Multivariate statistical analyses (PCA, PLS-DA, HCA) revealed distinct varietal and vintage-dependent clustering and identified key discriminant fatty acids, including linolenic acid (C18:3), lauric acid (C12:0), and arachidic acid (C20:0). These findings underscore the compositional diversity and functional potential of GP, reinforcing its suitability for applications in functional foods, nutraceuticals, and cosmetics, in alignment with circular economy principles.

## 1. Introduction

Grape pomace (GP), the solid residue after pressing grapes for juice or wine, is the main by-product of the world wine industry, generating around 20 million tons of waste per year. GP contains grape skins, seeds, stems, and pulp, and it accounts for approximately 20–25% of the original grape weight [1,2]. This biomass, which was often thrown out or utilized as low-value compost and animal feed, is now realized to be a source of bioactive compounds of immense value for the economy and the environment via its added value [3,4].

The principle of the circular economy is dedicated to the revalorization of waste with the production of added-value products, which has promoted research on the recycling of winemaking waste. Its use falls under sustainability strategies because it lessens wastage, makes better use of resources, and yields natural, health-promoting ingredients [5,6]. From the perspective of food science, GP is a rich and diversified source of bioactive compounds, such as phenolic compounds, flavonoids, anthocyanins, and lipophilic compounds with antioxidant, anti-inflammatory, antimicrobial, and cardioprotective activities [7,8]. In particular, GP antioxidants, such as polyphenols and anthocyanins, have garnered more and more attention due to their ability to neutralize reactive oxygen species (ROS), which lowers oxidative stress and helps prevent chronic illnesses like cancer, heart disease, and neurological disorders. Numerous in vitro and in vivo studies have demonstrated how polyphenols produced from grapes can improve endogenous antioxidant defenses, prevent lipid peroxidation, and modify important physiological signaling pathways. These substances’ antioxidant properties are also highly relevant for use in food and cosmetics, where they can act as anti-aging and natural preservatives, respectively. On the other hand, anthocyanin-containing pigmented GP of red grapes may serve as a natural colorant, antioxidant, or clean-label food additive. Its bioactivities, including free-radical scavenging and anti-inflammatory activity, are an object of the growing interest in health-promoting, sustainably sourced additives. Upcycling GP winemaking by-products to functional foods, edible coatings, and nutraceuticals enables industries to reduce waste while promoting environmentally friendly innovation and satisfying consumer demand for plant-based, minimally processed ingredients [7,9,10,11].

In line with sustainability and circular economic principles, the recovery of fatty acids from GP has become of vital interest. The bioactive compounds, derived primarily from the lipid fraction of the seeds, offer insight into the fatty acid composition and information on the functional and nutritional quality of GP oil. Therefore, the unsaturation ratio (USFA/SFA)—that is, the ratio of unsaturated to saturated fatty acids—is used to assess the food lipid fraction’s nutritional quality and estimate indices for the recurring syndromes of cardiovascular disease (e.g., thrombogenicity, atherogenicity) [12,13]. High values of this ratio are preferred in nutrition since they show how fatty acid affects cholesterol metabolism. A high H/H (hypocholesterolemic/hypercholesterolemic) ratio is considered favorable, as it reflects a higher proportion of fatty acids that help reduce LDL-cholesterol levels and improve lipid metabolism. Conversely, low values of the atherogenicity index (AI) and thrombogenicity index (TI) are desirable, as they indicate a lower risk of arterial plaque formation and thrombosis, respectively. The H/H ratio is associated with cholesterol metabolism, TI measures the risk of causing thrombus or blood clots [6], and AI measures the risk of artery blockage. High readings of this ratio indicate that oils are good for you since they help to enhance cardiovascular health [12]. These indices are widely used to evaluate the cardiovascular health potential of dietary fats and contribute to understanding the functional relevance of GP oil.

Extraction procedures such as Soxhlet [12,14,15], cold pressing [14], supercritical CO_2_ [16], and ultrasound-assisted extraction [6,15] have been evaluated for their potential to optimize the lipid yield, minimize solvent use, and preserve unsaturated fatty acids from winemaking by-products (e.g., grape seed). Soxhlet, the most commonly employed extraction procedure to isolate lipids from GP, takes long extraction times and consists of post-extraction concentration steps, which limit its environmental and operational efficiency. To overcome those limitations, simplified methods based on direct transesterification, such as that used in the present study, allow rapid and solvent-reduced profiling of FAMEs without prior lipid extraction. This not only lowers the processing time and environmental footprint but also complies with green chemistry principles and maximizes the opportunity for industrial upscaling of grape pomace valorization. The fatty acid profile of GP oil tends to have predominantly high levels of polyunsaturated fatty acids (PUFAs), especially linoleic acid (omega-6, ω-6), and monounsaturated fatty acids (MUFAs) like oleic acid, but with reduced proportions of saturated fatty acids. The ω-6-to-omega-3 (ω-3) fatty acid ratio and the degree of unsaturation are important indicators determining the nutritional value and food acceptability of the oil.

GP is used commercially for several purposes, including making brandies like the high-alcohol Portuguese beverage *aguardente bagaceira*. Additionally, it is employed in the production of fertilizers and substrates, as demonstrated by the Beifiur corporation, which operates research laboratories, a biofactory, and an organic fertilizer production line that develops biochar and microbial-based products from grape waste, supporting environmental and economic sustainability [17]. Portuguese businesses that use GP in novel ways include Soalheiro and Tintex, both of which are based in Alto Minho. They created a leather substitute fabric for apparel and bottle labels using organic cotton and grape pomace. Through a reduction in agricultural waste in the food and textile industries, this program fosters sustainability and creativity [1]. Current research has begun to explore the possibility of using grape seed or GP oils in food applications. Applications involve their utilization as edible oils, incorporation into salad dressings, and preparation of emulsion-based delivery systems for bioactive molecules. The inherent antioxidant profile of the oil may also confer oxidative stability, which would allow the shelf life of lipid-rich food products to be extended without using synthetic antioxidants. Moreover, the emulsifying properties of GP lipids make them ideal candidates for encapsulation technologies where they can act as polyphenol carriers, oil carriers, or fat-soluble vitamin carriers [9,18,19].

Despite its demonstrated potential, critical research gaps persist. Most studies lack integrated analyses of how the grape variety and vintage influence the GP composition and function. Comparative assessments of antioxidant and fatty acid profiles across multiple varieties and years are rare, as is their integration with functional indices relevant to food and cosmetic applications.

To address this gap, the present study aims to systematically compare the fatty acid profile, total phenolic content (TPC), total flavonoid content (TFC), and antioxidant activity (DPPH, ABTS, ORAC) of GP derived from seven *Vitis vinifera* L. varieties, across three consecutive vintages (2022–2024). The novelty lies in the standardized, comparative assessment of GP composition across multiple varieties and vintages, using a green, direct transesterification method. Unlike most existing studies that focus on either lipidomic or phenolic profiles in isolation, this study offers an integrated lipid–phenolic–antioxidant evaluation under uniform analytical conditions, which allows for robust cross-varietal comparisons and the identification of GP sources with enhanced multifunctional potential. This work provides the first comprehensive, side-by-side evaluation of GP’s multifunctional potential, supporting targeted valorization strategies in food and cosmetic applications aligned with circular economy principles.

## 2. Results and Discussion

### 2.1. Total Phenolics, Total Flavonoids, Total Anthocyanin, and Antioxidant Activity

The phenolic composition and antioxidant activity of GP from seven different grape varieties were assessed across three consecutive vintages (2022–2024) using seven in vitro assays: TPC, expressed as milligrams of gallic acid equivalent per 100 g, mgGAE/100 g; TFC, expressed as milligrams of quercetin equivalent per 100 g, mgQE/100 g; 2,2-diphenyl-1-picrylhydrazyl (DPPH), expressed as milligrams of Trolox equivalent per 100 g, mgTE/100 g; 2,2′-azino-bis(3-ethylbenzothiazoline-6-sulfonic acid) (ABTS), mgTE/100 g; and oxygen radical absorbance capacity (ORAC), expressed as micromole of Trolox equivalent per 100 g, µmolTE/100 g (Table 1). Significant variability was observed across grape varieties (*p* < 0.001) and vintages (*p* < 0.001 to *p* < 0.01) as well as between grape variety and vintage, reflecting both genotypic differences and the influence of annual climatic conditions, viticultural practices, and berry maturity on the biochemical composition of grape residues (Table S1).

TPC and TFC levels were highest in GP from Tinta Negra and Complexa across all vintages, with GP from Tinta Negra reaching 6687 mgGAE/100 g (TPC) and 4624 mgQE/100 g (TFC) in 2024, and Complexa reaching 6489 mgGAE/100 g (TPC) and 3985 mgQE/100 g (TFC) in 2023. These values consistently surpassed those of GP from white varieties (Boal, Malvasia, Sercial, Terrantez, Verdelho). No statistically significant differences in TPC were detected for Malvasia between 2023 and 2024, for Verdelho between 2023 and 2024, or for Complexa between 2022 and 2024 (*p* > 0.05). The present study quantified TPC in GP, revealing values ranging from 5873 to 6687 mgGAE/100 g in red grape GP and from 1334 to 2263 mgGAE/100 g in white grape GP. These values are consistent to those reported by other authors for ethanolic, methanolic, and acetone extracts of GP, where red GP (e.g., Cabernet Sauvignon, Tempranillo) typically exhibits TPC values in the range of 3500 to 7000 mgGAE/100 g, consistent with the high TPC found in Tinta Negra and Complexa. GP from white varieties, such as Fetească Albă and Tămâioasă Românească, often falls within 1000–2500 mgGAE/100 g, which matches the current results [1,10,20,21].

The results related to antioxidant activity assessed by the DPPH, ABTS, and ORAC assays are shown in Table 1. Complexa and Tinta Negra consistently exhibited the highest antioxidant activity across all three assays. For instance, Tinta Negra showed the maximum antioxidant capacity in 2024 for DPPH (3891 mgTE/100 g) and ABTS (6054 mgTE/100 g), while Complexa reached the highest DPPH value in 2023 (5399 mgTE/100 g) and ABTS (7219 mgTE/100 g). These findings align with the high TPC typically associated with pigmented grape varieties and emphasize their potential as valuable sources of natural antioxidants. These observations reinforce the potential of red GP as a candidate for valorization in food, cosmetic, or nutraceutical applications. In the current study, DPPH values for the investigated GPs ranged from 578 to 5399 mgTE/100 g, ABTS from 1538 to 7219 mgTE/100 g, and ORAC from 5553 to 18,471 µmolTE/100 g. These values are consistent with those previously observed in Montepulciano GP [22]. The elevated antioxidant activities in the studied GP may be attributed to the higher phenolic and flavonoid contents as well as the specific grape varieties and vintages analyzed. One-way ANOVA results revealed highly significant differences (*p* < 0.05) among GP from red and white varieties for the three in vitro assays: ABTS, DPPH, and ORAC (Table 1). In particular, the GP from Tinta Negra and Complexa consistently exhibited the highest antioxidant activity values across all three assays, significantly surpassing GP from white varieties (e.g., Boal, Malvasia, Sercial, Terrantez, and Verdelho). These results highlight the strong antioxidant potential of GP from Tinta Negra and Complexa, supporting their suitability for applications targeting oxidative stress reduction. Even though GPs from the white variety showed a low TPC, TFC, and antioxidant activity, they are not free from functional significance since those may be useful for applications where low oxidative reactivity, coloration, and a moderate sensory impact are required. They can be used as starting materials for products that call for high compositional stability without interference by polyphenolic chemical compounds, as excipient matrices in food or cosmetics, or in the preparation of dietary supplements with little taste. In formulations where antioxidant potential is not the first functional need, their simplicity may also provide easier standardization and blending [23].

### 2.2. Fatty Acid Profile of Grape Pomace

The fatty acid profile of GP is a crucial parameter for assessing its potential nutritional and health benefits. Previously, the analytical method used to quantify the fatty acids in GP was validated concerning selectivity, linearity, sensitivity, and precision. The results are summarized in Table 2. The method’s selectivity was determined by the nonappearance of interfering peaks at the retention time (RT) of the fatty acids studied. The linearity was evaluated in the concentration range of each fatty acid, with each compound exhibiting excellent correlation coefficients (r^2^ ≥ 0.993). The method ensured accurate measurement of small and large fatty acids in GP by presenting a wide dynamic range from as low as 0.58 mg/L for linoleic acid (C18:2) to 400 mg/L for oleic acid (C18:1). Determining the LOD and LOQ allowed the evaluation of the method’s sensitivity. C18:2 was the most sensitive (LOD: 0.03 mg/L, LOQ: 0.10 mg/L), while even higher-molecular-weight fatty acids such as behenic acid (C22:0) possessed LODs below 0.57 mg/L. Precision values were below the acceptable limit of 20%, and the majority were below 5%. Thus, based on these validation parameters, the method is suitably robust, precise, and sensitive for extensive fatty acid profiling in GP.

**Figure 1:**
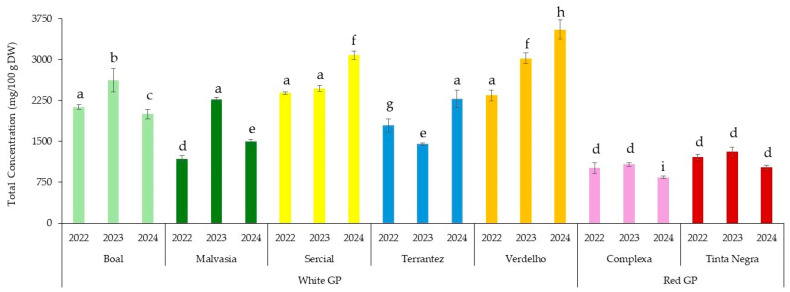
Total concentrations of fatty acids in the grape pomace from different grape varieties and vintages (2022–2024). Different letters indicate statistically significant differences between groups (*p* < 0.05), as determined by one-way ANOVA followed by Tukey’s HSD post hoc test.

Figure 1 shows the total fatty acids of the GP from different grape varieties and vintages. A two-way ANOVA showed that the grape variety had a significant effect on the total fatty acid content of GP (*p* < 0.001), as did the vintage year (*p* < 0.001). A significant interaction between grape variety and vintage was also observed (*p* < 0.001), indicating that the effect of the vintage on the fatty acid content varied depending on the grape variety. Post hoc analysis using Tukey’s HSD test (Table S3) revealed several statistically significant pairwise differences. For instance, GP from Boal 2022 differed significantly from GP from Complexa 2022 (mean difference = −1115.97, *p* < 0.001) and Tinta Negra 2023 (mean difference = −1211.01, *p* < 0.001), suggesting that red grape varieties generally exhibited lower total fatty acid contents than white varieties. In addition, the total fatty acids of the GP from Verdelho 2024, Boal 2023, Boal 2024, and Complexa 2024 were significantly different from the remaining GPs investigated (*p* < 0.05) (Figure 1). Moreover, statistical analysis (*p* < 0.05) indicated that the vintage significantly influenced the total concentrations of fatty acids for most of the GP investigated.

These results highlight the importance of both genetic and environmental factors in determining the lipid composition of GP, with implications for its targeted use in functional food and nutraceutical formulations. This finding is consistent with previous studies showing that vintage effects and specific climatic conditions (e.g., temperature, rainfall) were key drivers of variation in grape and wine composition, including phenolics and other metabolites [11,24].

Table 3 shows the fatty acid composition of the GP from different grape varieties and vintages. A consistent pattern related to major and minor fatty acids in GP was observed independently of grape variety or vintage.

Across all investigated GPs, C18:2 and C18:1 were the predominant fatty acids, averaging total contributions of 87 ± 2% and 92 ± 2% for total fatty acids for GPs from red and white grapes, respectively. These results are in agreement with the common fatty acid profile reported for grape seed oils from different *Vitis vinifera* L. grapes [6,25,26]. These major fatty acids showed considerable nutritional and health potential, since methyl linoleate, derived from linoleic acid (an essential omega-6 fatty acid), plays a crucial role in maintaining cell membrane integrity and regulating inflammatory responses [27,28]. Oleic acid is well-recognized for its cardiovascular benefits, including the reduction in LDL cholesterol and maintaining a healthy body weight [29]. The GPs from Verdelho, Sercial, and Boal generally exhibited the highest concentrations of these two fatty acids among the varieties studied, on average, a total of 2750 ± 474, 2463 ± 300, and 2070 ± 289 mg/100 g dw, respectively. The presence of these bioactive lipids in GPs underscores their potential as added-value by-products for nutraceutical, functional food, or cosmetic applications. Complexa and Tinta Negra exhibited significantly lower levels of C18:1 compared to Boal, Sercial, and Verdelho (*p* < 0.05). Additionally, Malvasia 2022, Malvasia 2024, Terrantez 2022, and Terrantez 2024 presented lower concentrations of C18:1n9 compared to Boal, Verdelho, and Sercial (*p* < 0.05). In addition, GP from Complexa showed a distinctive fatty acid profile, being the only GP containing detectable levels of C10:0 and C12:0. Moreover, C22:0 was only detected in GP from the Tinta Negra variety. The minor FAMEs, including methyl α-linolenoate, were detected in moderate amounts across all investigated GPs. Although it is lower in concentration, methyl α-linolenoate is of particular nutritional interest due to its origin from α-linolenic acid, known for its anti-inflammatory and cardioprotective properties [30,31].

**Figure 2:**
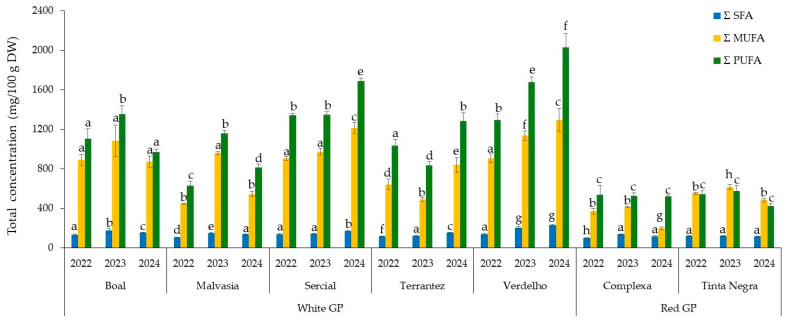
Total concentrations of monounsaturated (ΣMUFA), saturated (ΣSFA), and polyunsaturated (ΣPUFA) fatty acids in grape pomace from different grape varieties and vintages (2022–2024). Different letters within columns with the same color indicate statistically significant differences between groups (*p* < 0.05), as determined by one-way ANOVA followed by Tukey’s HSD post hoc test.

Figure 2 shows the distribution of fatty acids in GP from different grape varieties across vintages (2022–2024), revealing distinct trends. Polyunsaturated fatty acids (ΣPUFAs) consistently represented the predominant lipid class, followed by monounsaturated fatty acids (ΣMUFAs) and saturated fatty acids (ΣSFAs). The distribution of the fatty acids for GP from white grape varieties was on average 7 ± 1% and 93 ± 1% for saturated fatty acids (SFAs) and unsaturated fatty acids (UFAs), while for GP from red grape varieties, it was 11 ± 2% and 89 ± 2%, respectively. MUFAs corresponded to 39 ± 3% and 40 ± 8% for GP from white and red grape varieties, and PUFAs corresponded to 54 ± 2% and 49 ± 7%, respectively.

The application of direct FAME profiling, by passing the first Soxhlet extraction, allowed for the efficient quantitation of fatty acids with decreased solvent use and number of steps. This supports the feasibility for use in sustainable and scalable industrial processes. Interestingly, the total fatty acid contents produced by the direct transesterification method in this work are in agreement with literature values for Soxhlet-based extraction [12,13].

### 2.3. Multivariate Statistical Analysis

**Figure 3:**
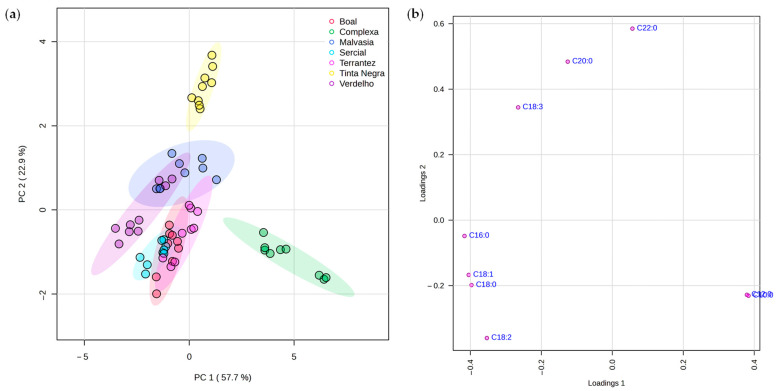
PCA of the fatty acid profiles of grape pomace from different grape varieties and vintages (n = 3 per data point): (**a**) score plot and (**b**) loading plot highlighting the contribution of individual fatty acids to PC1 and PC2.

A multivariate statistical analysis was carried out using the MetaboAnalyst 6.0 program to conduct the principal component analysis (PCA), partial least squares-discriminant analysis (PLS-DA), and hierarchical cluster analysis (HCA), as described in Section 3.7. The PCA score plot (Figure 3a) revealed a clear separation between GPs, highlighting inherent differences in fatty acid composition driven by both grape variety and vintage. PC1 and PC2 together explained a substantial portion of the total variance (80.6%), demonstrating the discriminative power of the dataset. The corresponding loading plot (Figure 3b) identified C18:1, C18:3, C12:0, and C16:0 as major contributors to this separation, as evidenced by their strong influence among the principal components.

**Figure 4:**
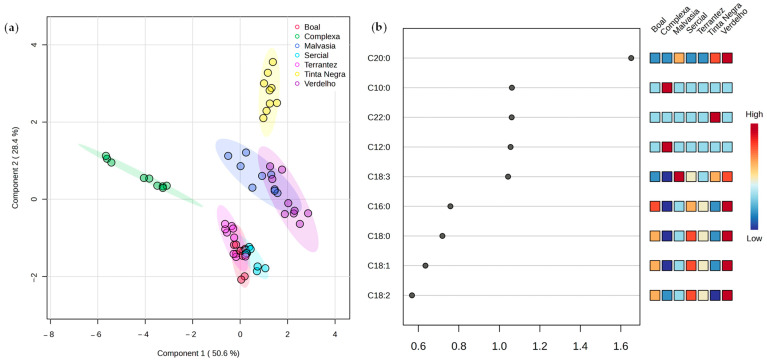
PLS-DA of the fatty acid profile of grape pomace from different grape varieties and vintages (*n* = 3 for each data point): (**a**) score scatter plot and (**b**) variable importance in projection (VIP) scores.

PLS-DA was performed to explore the potential of fatty acid composition to discriminate the GPs from different varieties and vintages. The score plot (Figure 4a) confirmed the differentiation among GPs, reinforcing varietal and vintage-dependent trends. The first two components obtained a total variance of 79% from PLS-DA (50.6% of Component 1 and 28.4% of Component 2). Variable importance in projection (VIP) analysis (Figure 4b) pinpointed key fatty acids, particularly C18:3, C12:0, C22:0, C10:0, and C20:0, as crucial variables with VIP scores exceeding the threshold of 1.0, suggesting their relevance as potential biomarkers for sample differentiation.

**Figure 5:**
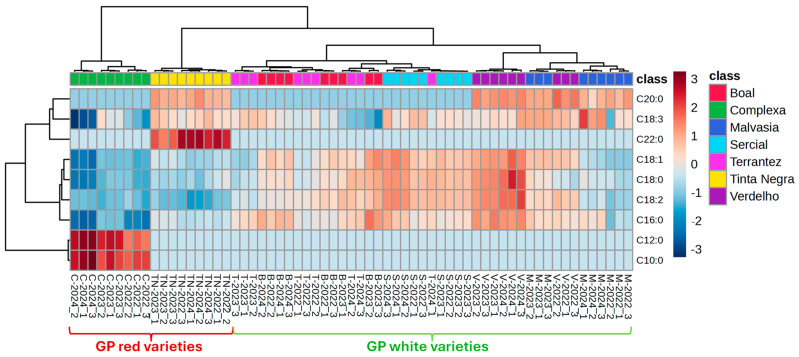
The average algorithm and Pearson distance analysis generated by HCA and heatmap of the investigated grape pomace. Samples are coded by grape variety (C—Complexa, TN—Tinta Negra, T—Terrantez, B—Boal, S—Sercial, V—Verdelho, M—Malvasia) and vintage (2022, 2023, 2024).

HCA combined with heatmap visualization (Figure 5) provided a complementary overview of the relationships among GPs. The clustering patterns aligned with those observed in PCA and PLS-DA, with clear grouping by grape variety and, to a lesser extent, by vintage. The Pearson correlation-based clustering underscored the compositional similarities among Terrantez and Boal varieties, while Malvasia and Tinta Negra showed more distinct profiles, particularly for samples from the 2024 vintage.

### 2.4. Functional Quality

The functional quality of GPs from different grape varieties across different vintages was evaluated based on several indicators, including the atherogenic index (AI), thrombogenic index (TI), hypocholesterolemic/hypercholesterolemic ratio (H/H), unsaturation index (UI), oxidative stability (COX), and PUFA/SFA ratio, and is presented in Table 4. These indicators provide insights into the health-promoting potential of GP as a functional food ingredient or nutraceutical resource.

The COX values, indicative of resistance to lipid peroxidation, range from 5.1 to 6.8, demonstrating that the investigated GPs showed the highest COX values (>5.0). This finding suggests greater oxidative stability of their lipid fractions, which is desirable for industrial and functional food applications. A one-way ANOVA followed by Tukey’s HSD test confirmed significant differences among the investigated GPs for COX values (*p* < 0.05), with Tinta Negra exhibiting significantly lower oxidative stability compared to other varieties. Moreover, the COX values show that Boal does not differ significantly from Malvasia, Verdelho, Terrantez, and Sercial (*p* < 0.05). The COX values obtained from GP under study are higher than those reported for olive oil (1.78) [32], palm oil (4.10), and canola oil (1.55) [33], similar to GP from five Spanish grape varieties’ oil [12] and sunflower oil (6.60) [33], and lower than that from flaxseed oils (10.8) [34].

The PUFA/SFA ratio is an important nutritional indicator, with higher values being desirable for cardiovascular health. In the present study, the PUFA/SFA ratio for all GPs investigated ranged from 3.6 to 9.8. According to the literature, a PUFA/SFA ratio greater than 0.45 is often recommended in the human diet [12]. The GPs from Sercial (9.5 ± 0.4) and Verdelho (8.8 ± 0.8) exhibited, on average, the highest PUFA/SFA ratio, significantly surpassing the GPs from Complexa and Tinta Negra (*p* < 0.05), highlighting their potentially superior lipid profiles for cardiovascular health. In contrast, Tinta Negra and Complexa presented the lowest PUFA/SFA ratios, significantly lower than those of GP from white varieties, suggesting a less favorable balance of unsaturated to saturated fatty acids. The PUFA/SFA ratio reported in the current study is quite similar to that reported for other GPs from white and red grape varieties [12,35].

The ω-6/ω-3 ratio, a key determinant in the inflammatory balance, varied substantially among the investigated GPs. For the investigated GPs, the ω-6/ω-3 ratio ranges from 13.7 to 81, lower than those reported for grape seed oils from Carbenet Sauvignon and Ives varieties (154 to 178), and consistent with that reported for GP from Aglianico Irpino (68.8) [35]. Tinta Negra showed the lowest ω-6/ω-3 ratio, significantly lower than those of GPs from Sercial and Verdelho (*p* < 0.05), suggesting a less favorable balance. No statistically significant differences were observed between Boal, Sercial, and Verdelho (*p* < 0.05), indicating a similar and more balanced fatty acid profile in these GP varieties.

The AI and TI are predictive of the potential risk associated with lipid consumption. AI is indicative of the potential for pro-atherogenic activity or anti-atherogenic effect through prevention of plaque by lowering cholesterol and phospholipid levels, respectively, and preventing coronary disease [36]. The TI quantitates the potential for clots to form in veins and arteries. As far as we know, there are no established official values for AI and TI, but in the literature, mostly food science and nutrition studies, general reference intervals or interpretation criteria are as follows: an AI or TI < 0.1 is excellent (highly cardioprotective), AI or TI 0.1–0.24 is good/acceptable, and AI or TI > 0.24 is less healthy [36,37]. All GP considered in the current study showed values of AI and TI lower than 0.24, suggesting an overall beneficial fatty acid profile with potential cardioprotective effects. These low indices are indicative of a reduced capacity to promote atheroma and thrombus formation, which aligns with dietary recommendations aimed at improving cardiovascular health. Among the GP varieties studied, Tinta Negra and Complexa consistently exhibited the highest values for both the AI and TI. A one-way ANOVA followed by Tukey’s HSD test confirmed significant differences between Tinta Negra, Verdelho, and Sercial (*p* < 0.05). The H/H ratio and unsaturation index (UI) further support these findings. Sercial and Verdelho demonstrated the highest H/H ratios (>22.5) and UI values (>150), which reflect a greater degree of unsaturated fatty acids, associated with improved fatty acid profiles and reduced cardiovascular risk.

Among the studied GPs, Sercial and Verdelho consistently had the best nutritional- and health-associated lipid profiles, with low AI and TI indexes, and high unsaturation and hypocholesterolemic activity. The results strongly confirm the valorization of the Sercial and Verdelho GPs as prospective agro-residues for the manufacture of functional foods, nutraceuticals, and lipid-enriched health supplements. Conversely, GPs from Complexa and Tinta Negra with lower nutritional contents may be more suitable for non-food applications unless the following processing improves their lipid quality.

## 3. Materials and Methods

### 3.1. Chemicals

All chemicals and reagents were of analytical quality grade. HPLC-grade methanol (MeOH), ethanol, and n-hexane were obtained from Fischer Scientific (Loughborough, UK). 2,2′-azinobis-(3-ethylbenzothiazoline-6-sulfonic acid) radical cation (ABTS^●^, 98%), Folin–Ciocalteu reagent (FR, 2N), sodium chloride (NaCl), Trolox (C_14_H_18_O_4_, 98%), sodium carbonate, (Na_2_CO_3_, 99,8%), 2,2-diphenyl-1-picrylhydrazyl (DPPH^●^, 90%), and acetyl chloride (CH_3_COCl, 98%) were purchased from Sigma-Aldrich (St. Louis, MO, USA). Aluminum chloride (AlCl_3_, 98%), potassium persulfate (K_2_S_2_O8, 99%), fluorescein, 2,2′-Azobis(2-amidinopropane) dihydrochloride (AAPH, ≥96%), potassium dihydrogen phosphate (KH_2_PO_4_), and sodium phosphate dibasic dodecahydrate (Na_2_PO_4_.12H_2_O) were acquired from Riedel-de Haën^®^ (Seelze, Germany). Solution Supelco 37 Components FAME (fatty acid methyl ester) Mix (Bellefonte, PA, USA), with a purity higher than 98%, was used to identify and quantify the FAMEs in GP under study by GC-FID. Ultrapure water (18 MΩ cm) obtained from a Milli-Q water purification system (Millipore, Milford, MA, USA) was used to prepare the aqueous solutions.

### 3.2. Samples

The grapes were harvested after ripening in the 2022, 2023, and 2024 vintages, and the pomace was kindly provided by Justino’s Madeira Wines S.A. (coordinates 32°39′04″ North latitude and 16°51′45″ West longitude) as a by-product of the winemaking process. For the current study, two red grape varieties (Complexa, Tinta Negra) and five white grape varieties (Boal, Malvasia, Terrantez, Verdelho, and Sercial) classified as noble grape varieties used to produce Maderia wine were taken into consideration. Table S1 shows the distribution of grape varieties across Madeira Island, revealing a predominance in the South and North regions, which together account for the majority of the total grape collection, with Tinta Negra (52.2% of total collection) and Terrantez (45.6%) being especially abundant in the South. The North region shows a significant contribution for Complexa (46.9%), Malvasia (99.4%), Sercial (62.4%), and Verdelho (71.6%). The West region also makes significant contributions, particularly for Boal (74.3%), while the East region contributes minimally to the overall grape product. Following pressing, using high-technology pneumatic presses once the grape is destemmed and crushed, without applying enzymatic preparations, the GP from each variety was gathered and brought to the lab in cool containers under refrigeration (2–5 °C). After being lyophilized (Telstar, Cryodos, Madrid, Spain) for ten hours, the GP was ground in a lab mill (Grindomix GM200, Rech, Germany) to obtain a fine, homogeneous powder and kept at −80 °C until analysis.

### 3.3. Total Phenolics, Total Flavonoids, and Antioxidant Activity

The total phenolic content (TPC), total flavonoid content (TFC), and antioxidant activity were determined in GP extracts obtained using a solution of 50:30:20 *v*/*v*/*v* of hexane, acetone, and ethanol. Briefly, 5 mL of 50:30:20 *v*/*v*/*v* hexane, acetone, and ethanol solution was added to a vial containing 0.5 g of lyophilized GP. After 10 min of ultrasonic agitation in an ultrasonic bath (Bransonic 2510), it was left at room temperature (25 ± 1 °C) for 2 h. The mixture was centrifuged for 5 min at 5000 rpm (centrifuge: SIGMA 1–7, St. Louis, MO, USA, maximum capacity 6 × 15 mL, maximum RCF 6153× *g*), which resulted in two distinct layers due to the immiscibility of hexane with the polar solvent mixture. The lower polar phase (acetone/ethanol-rich) was collected for subsequent determination of the TPC, TFC, and antioxidant activity using a UV-Vis spectrophotometer (Lambda 25, PerkinElmer, Waltham, MA, USA). All spectrometric assays were performed in triplicate.

The TPC was calculated spectrophotometrically using the Folin–Ciocalteu assay, while the TFC was estimated by the AlCl_3_ colorimetric assay, according to Abreu et al. [38]. The TPC was expressed as milligrams of gallic acid equivalent per 100 g of dry sample [mg(GAE)/100 g DW], while the TFC was expressed as milligrams of quercetin equivalent per 100 g of dry sample [mg(QE)/100 g DW], since gallic acid and quercetin were used as reference standards to plot the calibration curve, respectively. The antioxidant activity was assessed by in vitro assays, namely 2,2-diphenyl-1-picrylhydrazyl (DPPH), 2,2′-azino-bis(3-ethylbenzothiazoline-6-sulfonic acid) (ABTS), and oxygen radical absorbance capacity (ORAC) assays. The DPPH and ABTS assays were performed based on the procedure adopted by Izcara et al. [39]. The DPPH and ABTS results were expressed as milligrams equivalent of Trolox per 100 g dry weight of GP, mg(TE)/100 g DW. The ORAC assay was performed according to the protocol established by Och et al. [40], with some modifications. In a plate with 96 white flat-bottom wells, 150 µL of fluorescein (10 nM) and 25 µL of extract were added. The mixture was incubated for 20 min at 37 °C. Then, 25 µL of AAPH solution (240 nM) was added, and the fluorescence was read at 485 nm and 520 nm (excitation and emission wavelengths, respectively) in a Victor3 Multilabel Plate Counter 1420 fluorescence reader at 90 sec intervals until the fluorescence decayed and the absorbance became constant. The results were expressed in µmol(TE)/100 g DW. Phosphate buffer (75 mM, pH 7.4) was used as a blank. All antioxidant assays were performed in triplicate.

### 3.4. Preparation of Fatty Acid Extracts

The fatty acids were obtained through a trans-methylation reaction based on the protocol described by Ferreira et al. [13]. Briefly, 50 mg of lyophilized GP was suspending in 1 mL of hexane and 1 mL of transesterification reagent (5% MeOH: CH_3_COCl) in Pyrex tubes with screw caps. The tubes were vortexed for 10 sec and incubated in a thermostatic bath at 105 °C for 20 min. After cooling at room temperature (25 ± 1 °C), 1 mL of H_2_O was added to promote the separation of hydrophilic components. The mixture was centrifuged for 3 min at 3500 rpm to promote the separation of solutions into two phases. The upper solution was collected and injected into GC-FID. The derivatization process was carried out in triplicate.

### 3.5. GC-FID Conditions

The fatty acids were determined using a certified protocol established for standard solution Supelco 37 Components FAMEs Mix. An Agilent 7890A gas chromatograph (Agilent, Santa Clara, CA, USA) with a flame ionization detector (FID) and an Agilent 7693 autosampler were applied to achieve the fatty acids’ separation. The separation was achieved on an SPB-PUFA fused silica capillary column (30 m × 0.25 mm × 0.2 µm). The injection volume was 1 µL, using a split injection with a split ratio of 100:1, and helium was used as the carrier gas at a flow rate of 800 µL/min. The GC oven temperature started at 50 °C for 2 min, increased to 210 °C at 10 °C/min, and was held for 40 min, with a total runtime of 58 min. The injector temperature was 250 °C, and the FID temperature was 260 °C. Air and hydrogen were supplied to the FID at 450 and 40 mL/min flow rates, respectively. The fatty acid identification was performed by comparing their retention times to those of the standard solution, Supelco 37 Components FAMEs Mix, and the quantification was carried out through calibration curves by plotting the relative area vs. concentration. By injecting the identical standard solution containing fatty acids in triplicate on the same day (n = 9) and three consecutive days (n = 27), respectively, the intra- and inter-day accuracy was assessed, expressed as relative standard deviation (%RSD). The ratios of the standard deviation(s) of calibration curve interception, by the slope of a regression curve, were multiplied by 3 and 10 to calculate the limit of detection (LOD) and limit of quantification (LOQ), respectively. All analyses were performed in triplicate.

### 3.6. Functional Quality

The functional quality of GP from different grape varieties and vintages was assessed through the ratio between hypercholesterolemic fatty acids (H/H), the atherogenic index (AI), the thrombogenic index (TI), and oxidizability (COX) calculated based on the equations described by Ulbricht and Southgate [37] and Carmona-Jiménez [12].

### 3.7. Statistical Analysis

To ensure statistical significance, each experiment was performed in triplicate. The MetaboAnalyst 6.0 software [41] was used to evaluate the data using one-way analysis of variance (ANOVA) and Tukey’s multiple comparison test. A *p*-value of less than 0.05 was considered statistically significant for the investigated GPs from different grape varieties and vintages. PCA and PLS-DA were employed to provide insights into the separations among the GPs under study and to detect fatty acids that may represent differences among the sample sets. PLS-DA can reduce the size of the data matrix and remove extraneous variables to find fatty acid sets that best discriminate among the various GPs. GP variants were thought to be characterized by fatty acids that had a VIP score of 1 or above. Using the fatty acids found and measured in the GPs under investigation, HCA was carried out. The goal of this investigation was to find clustering patterns for the characterization of the GPs under study using the Euclidean distance and Ward’s method.

## 4. Conclusions

This work advances the understanding of GP’s compositional diversity across grape varieties from seven *Vitis vinifera* L. grapes and vintages (2022, 2023, and 2024) and substantiates its feasibility as a functional lipid ingredient for food and cosmetic applications. Thus, it contributes to the sustainable transformation of winery by-products into high-value resources.

Irrespective of vintage, GPs from Tinta Negra and Complexa consistently exhibited the highest TPC, TFC, and antioxidant activity, underscoring their strong potential for applications targeting oxidative stress mitigation. In contrast, fatty acid profiling revealed that C18:1 (oleic acid) and C18:2 (linoleic acid) were the predominant fatty acids, with their concentrations being markedly higher in GP from white grape varieties, particularly Verdelho and Sercial, compared to red varieties. GP from Verdelho and Sercial was characterized by elevated PUFA/SFA ratios (≥8.2), low AI (<0.05) and TI (≤0.13), high H/H ratios (>21), and increased oxidizability indices (UI and COX). These compositional attributes highlight GPs’ promise as sustainable ingredients for developing functional foods, nutraceuticals, and cosmetic products, thereby enabling the transformation of winery by-products into high-value bioactive materials with substantial health benefits. Nevertheless, it was also observed that the vintage significantly impacted the compositional profile of GP, namely the TPC, antioxidant activity, and fatty acid content. The findings confirm the significance of seasonality and climatic fluctuation in determining the functional and nutritional potential of GPs. This reinforces the need for future studies to include the vintage as a critical variable in GP valorization strategies.

The current study also demonstrates the analytical feasibility of direct FAME profiling without prior lipid extraction (e.g., Soxhlet), simplifying the process and supporting its scalability. The inclusion of multivariate statistical analyses (PCA, PLS-DA, HCA) provided deeper insight into varietal and vintage-driven variability, confirming compositional clustering and enabling the identification of fatty acid biomarkers, such as C18:3n3, C12:0, C22:0, C10:0, and C20:0, relevant to grape variety differentiation and targeted application development. Furthermore, the geographical origin of the grape varieties was clarified and systematically detailed, supporting traceability and reinforcing the relevance of terroir in shaping the biochemical attributes of GP. This data, paired with extensive compositional data, strengthens the foundation for industrial applications rooted in quality assurance and provenance. The comprehensive description of GP confirms that it is appropriate for high-value circular economic initiatives. White GP may be preferred for cardioprotective formulations because of its superior lipid indices, whereas GP from red varieties, which is rich in phenolic antioxidants, may be given priority for applications that address oxidative stress. These results open the door for future pilot-scale and industrial applications and support the development of customized valorization methods for GP.

Future research should prioritize comprehensive toxicological, stability, and bioavailability assessments to ensure the safety and efficacy of GP-derived products intended for human consumption or topical application.

## Figures and Tables

**Table 1:** Total phenolic content (TPC), total flavonoid content (TFC), and antioxidant capacity (DPPH, ABTS, ORAC) of GP from different grape varieties and vintages. The values are expressed as mean of concentration (mg) ± SD per 100 g of dry weight (DW).

Grape Pomace	Vintage	TPC (mgGAE/100 g)	TFC (mgQE/100 g)	DPPH (mgTE/100 g)	ABTS (mgTE/100 g)	ORAC (µmolTE/100 g)
Boal	2022	2263 ± 15 ^a^	851 ± 28 ^a^	984 ± 25 ^a^	2275 ± 124 ^a^	9742 ± 540 ^a^
	2023	1722 ± 21 ^b^	1064 ± 23 ^b^	1284 ± 2 ^a,b^	2453 ± 63 ^a,b^	8935 ± 23 ^a,g^
	2024	1416 ± 17 ^c^	734 ± 28 ^a^	1009 ± 52 ^a^	2160 ± 146 ^a,c^	9054 ± 711 ^a,g^
Malvasia	2022	1902 ± 5 ^b^	865 ± 12 ^a^	1167 ± 22 ^a,b^	2894 ± 38 ^b^	9685 ± 77 ^a,g^
	2023	1489 ± 14 ^c^	824 ± 16 ^a^	876 ± 16 ^b,c^	1752 ± 31 ^a,c^	6955 ± 10 ^b^
	2024	1504 ± 6 ^c^	928 ± 21 ^a,b^	1027 ± 40 ^a,b,c^	2354 ± 35 ^a,b,c^	6340 ± 45 ^b^
Sercial	2022	1984 ± 5 ^d^	995 ± 9 ^a,b^	1098 ± 33 ^a,b^	2668 ± 99 ^a,b,c^	8751 ± 302 ^a,g^
	2023	1729 ± 4 ^b^	773 ± 16 ^a^	578 ± 1 ^c^	1887 ± 27 ^c^	8662 ± 226 ^a,g^
	2024	2051 ± 6 ^d^	671 ± 20 ^a^	794 ± 84 ^a,c^	1538 ± 27 ^d^	8697 ± 423 ^a,g^
Terrantez	2022	1834 ± 2 ^b,d^	690 ± 1 ^a^	772 ± 19 ^a^	1711 ± 90 ^c^	5965 ± 174 ^b^
	2023	1650 ± 7 ^b,e^	876 ± 41 ^a,b^	1074 ± 28 ^a,b^	2374 ± 30 ^a^	6977 ± 251 ^b,g^
	2024	1696 ± 3 ^b,e^	937 ± 3 ^a,b^	1291 ± 22 ^a,b^	2827 ± 16 ^g^	5553 ± 37 ^b^
Verdelho	2022	1354 ± 21 ^c^	933 ± 33 ^a,b^	854 ± 14 ^a^	2106 ± 118 ^a^	8375 ± 180 ^a,g^
	2023	1853 ± 10 ^b,d^	825 ± 19 ^a^	1241 ± 39 ^a,b^	2673 ± 147 ^a,b^	7620 ± 534 ^c,g^
	2024	1856 ± 8 ^b,d^	904 ± 2 ^a,b^	1295 ± 20 ^a,b^	2674 ± 214 ^a,b^	8160 ± 240 ^c,g^
Complexa	2022	5972 ± 189 ^h^	3628 ± 235 ^f^	3565 ± 168 ^f^	6102 ± 53 ^h^	15,866 ± 665 ^d^
	2023	6489 ± 97 ^i^	3985 ± 104 ^g^	5399 ± 172 ^g^	7219 ± 104 ^i^	16,576 ± 1177 ^d^
	2024	5873 ± 7 ^h^	3233 ± 35 ^h^	3881 ± 346 ^f^	5460 ± 732 ^j^	15,870 ± 378 ^d^
Tinta Negra	2022	6606 ± 62 ^i^	4156 ± 25 ^g^	3502 ± 142 ^f^	5849 ± 94 ^h,j^	18,471 ± 653 ^e^
	2023	6084 ± 178 ^h^	3812 ± 7 ^g^	3294 ± 80 ^i^	4848 ± 210 ^k,j^	14,215 ± 229 ^f^
	2024	6687 ± 32 ^i^	4624 ± 55 ^i^	3891 ± 89 ^f^	6054 ± 268 ^h,j^	16,692 ± 68 ^d^

Different letters within each column indicate statistically significant differences between groups (*p* < 0.05), as determined by one-way ANOVA followed by Tukey’s HSD post hoc test.

**Table 2:** Figures of merit of the method.

RT (min)	FAME	Concentration Range (mg/L)	r^2^	Equation	LOD (mg/L)	LOQ (mg/L)	Precision (%RSD)	
							Intra-Day	Inter-Day
14.09	Decanoic acid, C10:0	3.58–100	0.998	y = 0.106x + 0.752	0.85	2.83	2.46	14.2
17.44	Lauric acid, C12:0	3.56–100	0.999	y = 0.124x + 0.526	0.39	1.30	1.52	4.74
21.96	Palmitic acid, C16:0	5.43–100	0.999	y = 0.179x – 0.338	0.32	1.07	0.65	1.57
26.23	Stearic acid, C18:0	3.56–100	0.999	y = 0.164x – 0.293	0.34	1.14	1.03	6.43
26.63	Oleic acid, C18:1	3.56–400	0.999	y = 0.268x – 0.282	0.22	0.74	1.62	9.68
27.81	Linoleic acid, C18:2	0.58–300	0.998	y = 1.043x – 5.658	0.03	0.10	2.82	9.86
29.84	Linolenic acid, C18:3	1.73–200	0.996	y = 0.213x – 0.332	0.16	0.52	1.16	6.47
33.42	Arachidic acid, C20:0	3.56–39.6	0.994	y = 0.194x + 0.197	0.12	0.40	1.23	11.7
47.53	Behenic acid, C22:0	3.55–39.4	0.993	y = 0.164x + 1.386	0.57	1.91	2.12	5.99

**Table 3:** Fatty acid profile (mg/100 g of dried sample ± standard deviation) of grape pomace from different *Vitis vinifera* L. grapes.

Grape Pomace	Vintage	Concentration (mg/100 g DW) ± Standard Deviation								
		C10:0	C12:0	C16:0	C18:0	C18:1	C18:2	C18:3	C20:0	C22:0
Boal	2022	-	-	96 ± 9 ^a^	36 ± 2 ^a^	888 ± 56 ^a^	1081 ± 101 ^a^	24 ± 2 ^a^	-	-
	2023	-	-	126 ±17 ^b^	49 ± 6 ^a,b^	1084 ± 160 ^a^	1339 ±80 ^b^	17 ± 2 ^a,b^	-	-
	2024	-	-	119 ± 2 ^b,c^	37 ± 3 ^a,b^	875 ± 56 ^a^	944 ± 34 ^a^	26 ± 2 ^a^	-	-
Malvasia	2022	-	-	73 ± 7 ^d^	24 ± 2 ^c^	448 ± 2 ^b^	605 ± 46 ^c^	23 ± 4 ^a,b^	10 ± 1 ^a^	-
	2023	-	-	103 ± 4 ^a^	37 ± 3 ^b,d^	959 ± 14 ^a^	1127 ± 29 ^b^	33.1 ± 0.1 ^c^	9.6 ± 0.1 ^a^	
	2024	-	-	100 ± 4 ^a^	29 ± 2 ^a,c^	546 ± 28 ^b^	774 ± 35 ^d^	37 ± 4 ^d^	7 ± 1 ^b^	-
Sercial	2022	-	-	95 ± 3 ^a^	47 ± 1 ^a,b^	904 ± 18 ^a^	1312 ± 16 ^a,b^	29 ± 2 ^a^	-	-
	2023	-	-	97 ± 4 ^a^	48 ± 1 ^a,b^	973 ± 34 ^a^	1325 ± 25 ^a,b^	26 ± 1 ^a^	-	-
	2024	-	-	114 ± 6 ^a,b^	60 ± 1 ^b^	1213 ± 57 ^c^	1663 ± 28 ^e^	28 ± 3 ^a^	-	-
Terrantez	2022	-	-	88 ± 3 ^a^	29 ± 2 ^a,c,d^	642 ± 54 ^b^	1009 ± 62 ^a^	24 ± 1 ^a,b,c^	-	-
	2023	-	-	101 ± 5 ^a^	24 ± 2 ^a,c^	491 ± 21 ^b^	805 ± 44 ^d^	28 ± 2 ^a,c^	-	-
	2024	-	-	106 ± 2 ^a,b^	50 ± 1 ^b^	838 ± 74 ^a^	1265 ± 80 ^a^	20 ± 3 ^a,b,c^	-	-
Verdelho	2022	-	-	99 ± 4 ^a^	26 ± 3 ^a,c,d,^	908 ± 41 ^a^	1264 ± 61 ^a,b^	30 ± 1 ^a,c^	14 ± 2 ^c^	-
	2023	-	-	132 ± 7 ^b^	62 ± 2 ^b^	1135 ± 47 ^c^	1652 ± 51 ^e^	26 ± 1 ^a,c^	12 ± 1 ^a^	-
	2024	-	-	132 ± 3 ^b^	84 ± 10 ^b^	1294 ± 116 ^c^	1997 ± 144 ^f^	29 ± 3 ^a,c^	12 ± 1 ^a^	-
Complexa	2022	17 ± 1 ^a^	23 ± 2 ^a^	44 ± 2 ^f^	15 ± 2 ^c^	371 ± 28 ^b^	524 ± 90 ^c^	17 ± 2 ^a,b^	-	-
	2023	19 ± 3 ^a^	37 ± 2 ^b^	64 ± 1 ^g^	17 ± 1 ^c^	413 ± 7 ^b^	500 ± 35 ^c^	25 ± 2 ^a,c^	-	-
	2024	28 ± 4 ^c^	46 ± 4 ^b^	38 ± 1 ^f^	7.1 ± 0.5 ^f^	201 ± 14 ^h^	513 ± 11 ^c^	10 ± 1 ^b,f^	-	-
Tinta Negra	2022	-	-	78 ± 2 ^g,i^	26 ± 5 ^a,c,d^	552 ± 9 ^b^	517 ± 33 ^c^	26 ± 1 ^a,c^	8.0 ± 0.3 ^a^	9 ± 1 ^a^
	2023	-	-	85 ± 1 ^a,i^	22 ± 1 ^b,c^	617 ± 29 ^b^	546 ± 56 ^c^	29 ± 2 ^a,c^	10 ± 1 ^a^	5 ± 1 ^b^
	2024	-	-	78 ± 2 ^g,i^	21 ± 3 ^c^	483 ± 15 ^b^	397 ± 23 ^c^	29 ± 2 ^a,c^	10 ± 1 ^a^	9 ± 1 ^a^

Different letters within each column indicate statistically significant differences between groups (*p* < 0.05), as determined by one-way ANOVA followed by Tukey’s HSD post hoc test.

**Table 4:** Indicator of nutritional quality of the grape pomace from different grape varieties.

GP	Vintage	COX	PUFA/SFA	ω-6/ω-3	AI	TI	H/H	UI
Boal	2022	5.9 ± 0.3 ^a^	8 ± 1 ^a^	45 ± 8 ^a^	0.05 ± 0.01 ^a^	0.13 ± 0.01 ^a^	21 ± 2 ^a^	147 ± 4 ^a^
	2023	5.9 ± 0.4 ^a^	8 ± 1 ^a^	81 ± 15 ^b^	0.05 ± 0.01 ^a^	0.14 ± 0.01 ^a^	20 ± 2 ^a^	146 ± 4 ^a,b^
	2024	5.6 ± 0.1 ^b^	6.2 ± 0.2 ^b^	37 ± 4 ^a,c^	0.065 ± 0.004 ^a,b^	0.16 ± 0.01 ^b^	16 ± 1 ^b^	142.0 ± 0.4 ^a,b^
Malvasia	2022	6.1 ± 0.2 ^a^	5.9 ± 0.2 ^c^	27 ± 3 ^c^	0.067 ± 0.004 ^a,b^	0.161 ± 0.002 ^b^	15 ± 1 ^b^	146 ± 2 ^a,b^
	2023	5.85 ± 0.05 ^a^	7.8 ± 0.5 ^a^	34.1 ± 0.9 ^a,c^	0.049 ± 0.003 ^a,b^	0.12 ± 0.01 ^a^	21 ± 1 ^a^	146 ± 1 ^a,b^
	2024	6.2 ± 0.1 ^a^	5.9 ± 0.1 ^c^	21 ± 2 ^e^	0.074 ± 0.002 ^a,b^	0.168 ± 0.003 ^b^	13.5 ± 0.3 ^b^	148 ± 1 ^a,b^
Sercial	2022	6.31 ± 0.05 ^a,b^	9.5 ± 0.4 ^a^	45 ± 2 ^a^	0.042 ± 0.002 ^a,c^	0.118 ± 0.005 ^a^	24 ± 1 ^a^	152 ± 1 ^a,b^
	2023	6.15 ± 0.02 ^a,b^	9.3 ± 0.3 ^a^	52 ± 2 ^a^	0.042 ± 0.003 ^a,c^	0.12 ± 0.01 ^a^	24 ± 1 ^a^	149.9 ± 0.2 ^a,b^
	2024	6.2 ± 0.1 ^a,b^	9.8 ± 0.4 ^a^	61 ± 7 ^d^	0.039 ± 0.003 ^a,c^	0.11 ± 0.01 ^a^	26 ± 2 ^c^	150.2 ± 0.5 ^a,b^
Terrantez	2022	6.5 ± 0.1 ^a,b^	8.8 ± 0.3 ^a^	42.0 ± 0.8 ^a,c^	0.053 ± 0.002 ^a,b^	0.131 ± 0.004 ^a^	19 ± 1 ^d^	152 ± 1 ^a,b^
	2023	6.5 ± 0.2 ^a,b^	6.7 ± 0.6 ^b,c^	28 ± 3 ^a,c^	0.076 ± 0.004 ^a,b^	0.17 ± 0.01 ^b^	13 ± 1 ^b^	151 ± 3 ^a,b^
	2024	6.28 ± 0.02 ^a,b^	8.3 ± 0.7 ^a^	64 ± 5 ^d^	0.050 ± 0.005 ^a,b^	0.14 ± 0.01 ^a^	20 ± 2 ^a^	150.4 ± 0.3 ^a,b^
Verdelho	2022	6.2 ± 0.1 ^a,b^	9.3 ± 0.1 ^a^	42 ± 1 ^a^	0.045 ± 0.001 ^a,c^	0.106 ± 0.001 ^a^	22.2 ± 0.3 ^a^	150.6 ± 0.7 ^a,b^
	2023	6.20 ± 0.03 ^a,b^	8.2 ± 0.4 ^a,b^	63 ± 2 ^d^	0.047 ± 0.003 ^a,c^	0.13 ± 0.01 ^a^	21 ± 1 ^a^	150 ± 1 ^a,b^
	2024	6.3 ± 0.2 ^a,b^	8.9 ± 0.7 ^a^	70 ± 3 ^b,d^	0.040 ± 0.002 ^a,c^	0.125 ± 0.005 ^a^	25 ± 1 ^e^	151 ± 3 ^a,b^
Complexa	2022	6.04 ± 0.3 ^a^	5.5 ± 0.8 ^b,c^	30 ± 2 ^a,c^	0.07 ± 0.01 ^a,b^	0.12 ± 0.01 ^a^	21 ± 2 ^a^	145 ± 4 ^a,b^
	2023	5.68 ± 0.1 ^a,b^	3.8 ± 0.2 ^d^	20 ± 3 ^e^	0.108 ± 0.003 ^a,b^	0.152 ± 0.001 ^b^	15 ± 1 ^b^	138.4 ± 1.6 ^b,c^
	2024	6.8 ± 0.1 ^b^	4.4 ± 0.3 ^b,d^	50 ± 4 ^a,c^	0.12 ± 0.01 ^a,b^	0.12 ± 0.01 ^a^	19 ± 1 ^a^	149 ± 1 ^a,b^
Tinta Negra	2022	5.3 ± 0.1 ^f^	4.5 ± 0.1 ^b,d^	19.6 ± 0.3 ^e^	0.071 ± 0.002 ^a,b^	0.17 ± 0.01 ^b^	14.0 ± 0.4 ^b^	137 ± 1 ^c^
	2023	5.2 ± 0.2 ^f^	4.7 ± 0.4 ^b,d^	19 ± 1 ^e^	0.072 ± 0.004 ^a,b^	0.16 ± 0.01 ^b^	14 ± 1 ^b^	137 ± 2 ^c^
	2024	5.1 ± 0.1 ^f^	3.6 ± 0.2 ^d^	13.7 ± 0.1 ^e^	0.086 ± 0.002 ^a,b^	0.19 ± 0.01 ^b^	11.6 ± 0.2 ^b^	133 ± 1 ^c^

Different letters within each column indicate statistically significant differences between groups (*p* < 0.05), as determined by one-way ANOVA followed by Tukey’s HSD post hoc test.

## Data Availability

The original contributions presented in this study are included in the article/Supplementary Materials, and further inquiries can be directed to the corresponding author.

## Supplementary Materials

The following supporting information can be downloaded at https://www.mdpi.com/article/10.3390/molecules30153150/s1, Figure S1. Most representative geographic location of *Vitis vinifera* L. grape production on Madeira Island. Table S1. Distribution of grape varieties collected across Madeira Island, grouped into four geographic regions: North, South, East, and West. Data is presented in a number of grapevine units in kilograms. Table S2. Two-way ANOVA summary for total phenolic content (TPC), total flavonoid content (TFC), total carotenoids (TCs), and antioxidant capacity (DPPH, ABTS, ORAC) of grape pomace from different grape varieties and vintages. Table S3. Two-way ANOVA with post hoc Tukey HSD test (*p* < 0.01) of total fatty acid composition.

## Abbreviations

The following abbreviations are used in this manuscript:

ABTS	2,2′-azinobis-(3-ethylbenzothiazoline-6-sulfonic acid)
AI	Atherogenic index
C10:0	Decanoic acid
C12:0	Lauric acid
C16:0	Palmitic acid
C18:0	Stearic acid
C18:1	Oleic acid
C18:2	Linoleic acid
C18:3	Linolenic acid
C20:0	Arachidic acid
C22:0	Behenic acid
C3GE	Cyanidin-3-glucoside equivalents
COX	Oxidative stability
DPPH	1,1-diphenyl-2-picrylhydrazyl
DW	Dry weight
FAMEs	Fatty acid methyl esters
GAE	Gallic acid equivalent
GP	Grape pomace
H/H	Hypocholesterolemic/hypercholesterolemic ratio
MUFAs	Monounsaturated fatty acids
mgGAE	Milligrams of gallic acid equivalent
mgQE	Milligrams of quercetin equivalent
mgC3GE	Milligrams of cyanidin-3-glucoside equivalent
mgTE	Milligrams of Trolox equivalent
µmolTE	Micromoles of Trolox equivalent
ORAC	Oxygen radical absorbance capacity
LOD	Limit of detection
LOQ	Limit of quantification
PBS	Phosphate-buffered saline
PUFAs	Polyunsaturated fatty acids
QE	Quercetin equivalent
%RSD	Percentage of relative standard deviation
SFA	Saturated fatty acid
UI	Unsaturation index
TE	Trolox equivalent
TFC	Total flavonoid content
TI	Thrombogenic index
TPC	Total phenolic content
