# Supplementary material for: Unlocking the Fatty Acid and Antioxidant Profile of Grape Pomace: A Systematic Assessment Across Varieties and Vintages for Its Sustainable Valorization
Source: Molecules. 2025 Jul 28;30(15):3150. doi: 10.3390/molecules30153150 (PMC12348267; doi:10.3390/molecules30153150)
Supplement: Supplementary file 1 [file molecules-30-03150-s001.zip › molecules-3656027-supplementary.pdf]

Article

Unlocking the Fatty Acid and Antioxidant Profile of Grape Pomace: A Systematic Assessment Across Varieties and Vintages for Its Sustainable Valorization

Teresa Abreu [1], Rui Ferreira [1], Paula C. Castilho [1], José S. Câmara [1,2], Juan Teixeira [3] and Rosa Perestrelo [1,*]

[1] CQM—Centro de Química da Madeira, Universidade da Madeira, Campus da Penteada, 9020-105 Funchal, Portugal; teresa.abreu@staff.uma.pt (T.A.); rui.ferreira@staff.uma.pt (R.F.); pcastilho@staff.uma.pt (P.C.C.); jsc@staff.uma.pt (J.S.C.)

[2] Departamento de Química, Faculdade de Ciências Exatas e Engenharia, Universidade da Madeira, Campus da Penteada, 9020-105 Funchal, Portugal

[3] Parque Industrial da Cancela, Justino´s Madeira Wines, S.A., Caniço, 9125-042 Santa Cruz, Portugal; juan.teixeira@justinosmadeira.com

* Correspondence: rmp@staff.uma.pt

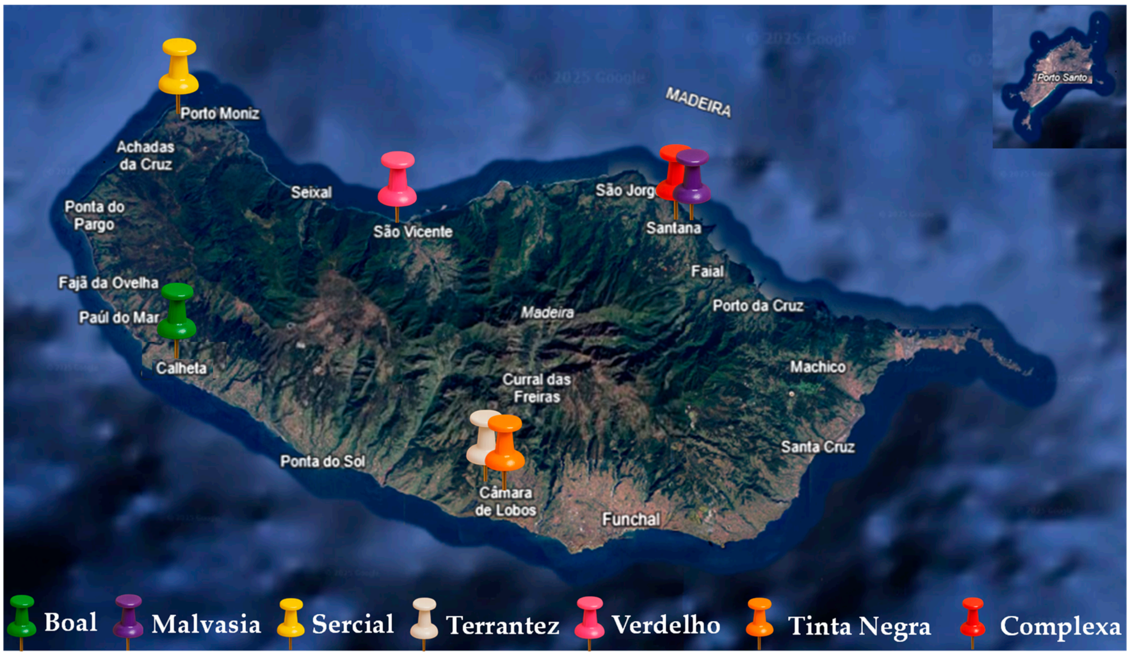

Figure S1. Most representative geographic location of *Vitis vinifera* L. grapes production in Madeira Island.

Table S1. Distribution of grape varieties collected across Madeira Island, grouped into four geographic regions: North, South, East, and West. Data is presented in number of grapevine units in kilograms.

**Madeira Island (Locals)**	**Boal**	**Malvasia**	**Sercial**	**Terrantez**	**Verdelho**	**Complexa**	**Tinta Negra**
				**North**			
Santana	2 562	**151 829**	4 984	1 490	8 284	**23 659**	5
São Vicente	382	4 907	10 156	17	**105 710**	2 153	1 149 422
Porto Moniz	70	0	**43 771**	1 117	52 856	1 046	0
**Sub-total**	3014	156736	58 911	2 624	166 850	26 858	1149427
%	**2.86%**	**99.4%**	**62.8%**	**11.1%**	**71.6%**	**46.9%**	**46,6%**
				**South**			
Câmara de Lobos	12 890	0	33 994	**10 424**	35 038	15 634	**1 287 126**
Funchal	10 960	0	0	400	440	1 560	0
**Sub-total**	23 850	0	33994	10824	35478	17 194	1287126
%	**22.6%**	**0%**	**36.3%**	**45.6%**	**15.2%**	**30.0%**	**52.2%**
				**East**			
Machico	0	916	0	0	876	0	0
Santa Cruz	173	0	69	2 866	1 703	459	0
Porto Santo	30	0	0	0	0	0	0
**Sub-total**	203	916	69	2 866	2 579	459	0
%	**0.19%**	**0.58%**	**0.07%**	**12.1%**	**1.1%**	**0.8%**	**0%**
				West			
Calheta	**67 640**	0	363	6 821	25 489	11 640	0
Ponta do Sol	0	0	0	524	1 865	0	0
Ribeira Brava	10 777	0	413	80	661	1 097	29 753
**Sub-total**	78 417		776	7425	28015	12 737	29 753
%	**74.3%**	**0%**	**0.8%**	**31.3%**	**12.0%**	**22.3%**	**1.2%**
**Total**	**105 484**	**157 652**	**93 750**	**23 739**	**232 922**	**57 248**	**2 466 306**

Table S2. Two-Way ANOVA summary for total phenolic content (TPC), total flavonoid content (TFC), and antioxidant capacity (DPPH, ABTS, ORAC) of grape pomace from different grape varieties and vintages.

Variable	Source of Variation	Degrees of Freedom	F-value	p-value
TPC	C(GP)	6	1.089e+04	2.32e-65
TPC	C(Vintage)	2	25.68	5.194e-08
TPC	C(GP):C(Vintage)	12	65.38	3.431e-23
TFC	C(GP)	6	5257	1.005e-58
TFC	C(Vintage)	2	0.4786	0.623
TFC	C(GP):C(Vintage)	12	50.13	6.106e-21
DPPH	C(GP)	6	1692	2.054e-48
DPPH	C(Vintage)	2	33.41	2.075e-09
DPPH	C(GP):C(Vintage)	12	53.74	1.59e-21
ABTS	C(GP)	6	735.7	7.165e-41
ABTS	C(Vintage)	2	0.8587	0.431
ABTS	C(GP):C(Vintage)	12	29.44	1.38e-16
ORAC	C(GP)	6	668.1	5.299e-40
ORAC	C(Vintage)	2	27.47	2.349e-08
ORAC	C(GP):C(Vintage)	12	14.17	3.966e-11

Table S3. Two-way ANOVA with post-hoc Tukey HSD ($p < 0.01$) of total fatty acids composition

group1	group2	meandiff	p-adj	lower	upper	reject
Boal_2022	Boal_2023	488.1967	0.0011	126.5084	849.8849	True
Boal_2022	Boal_2024	-126.4433	0.9984	-488.1316	235.2449	False
Boal_2022	Complexa_2022	-1115.9733	0.0	-1477.6616	-754.2851	True
Boal_2022	Complexa_2023	-1051.97	0.0	-1413.6583	-690.2817	True
Boal_2022	Complexa_2024	-1284.6267	0.0	-1646.3149	-922.9384	True
Boal_2022	Malvasia_2022	-944.5567	0.0	-1306.2449	-582.8684	True
Boal_2022	Malvasia_2023	142.4167	0.9935	-219.2716	504.1049	False
Boal_2022	Malvasia_2024	-633.1133	0.0	-994.8016	-271.4251	True
Boal_2022	Sercial_2022	260.54	0.4475	-101.1483	622.2283	False
Boal_2022	Sercial_2023	342.65	0.0819	-19.0383	704.3383	False
Boal_2022	Sercial_2024	950.9167	0.0	589.2284	1312.6049	True
Boal_2022	Terrantez_2022	-334.5767	0.1	-696.2649	27.1116	False
Boal_2022	Terrantez_2023	-677.7533	0.0	-1039.4416	-316.0651	True
Boal_2022	Terrantez_2024	152.4333	0.9862	-209.2549	514.1216	False
Boal_2022	Tinta Negra_2022	-910.9833	0.0	-1272.6716	-549.2951	True
Boal_2022	Tinta Negra_2023	-812.47	0.0	-1174.1583	-450.7817	True
Boal_2022	Tinta Negra_2024	-1100.0933	0.0	-1461.7816	-738.4051	True
Boal_2022	Verdelho_2022	214.5733	0.7696	-147.1149	576.2616	False
Boal_2022	Verdelho_2023	893.1667	0.0	531.4784	1254.8549	True
Boal_2022	Verdelho_2024	1421.2933	0.0	1059.6051	1782.9816	True
Boal_2023	Boal_2024	-614.64	0.0	-976.3283	-252.9517	True
Boal_2023	Complexa_2022	-1604.17	0.0	-1965.8583	-1242.4817	True
Boal_2023	Complexa_2023	-1540.1667	0.0	-1901.8549	-1178.4784	True
Boal_2023	Complexa_2024	-1772.8233	0.0	-2134.5116	-1411.1351	True
Boal_2023	Malvasia_2022	-1432.7533	0.0	-1794.4416	-1071.0651	True
Boal_2023	Malvasia_2023	-345.78	0.0757	-707.4683	15.9083	False
Boal_2023	Malvasia_2024	-1121.31	0.0	-1482.9983	-759.6217	True
Boal_2023	Sercial_2022	-227.6567	0.6825	-589.3449	134.0316	False
Boal_2023	Sercial_2023	-145.5467	0.9916	-507.2349	216.1416	False
Boal_2023	Sercial_2024	462.72	0.0025	101.0317	824.4083	True

Boal_2023	Terrantez_2022	-822.7733	0.0	-1184.4616	-461.0851	True
Boal_2023	Terrantez_2023	-1165.95	0.0	-1527.6383	-804.2617	True
Boal_2023	Terrantez_2024	-335.7633	0.0972	-697.4516	25.9249	False
Boal_2023	Tinta Negra_2022	-1399.18	0.0	-1760.8683	-1037.4917	True
Boal_2023	Tinta Negra_2023	-1300.6667	0.0	-1662.3549	-938.9784	True
Boal_2023	Tinta Negra_2024	-1588.29	0.0	-1949.9783	-1226.6017	True
Boal_2023	Verdelho_2022	-273.6233	0.3614	-635.3116	88.0649	False
Boal_2023	Verdelho_2023	404.97	0.0148	43.2817	766.6583	True
Boal_2023	Verdelho_2024	933.0967	0.0	571.4084	1294.7849	True
Boal_2024	Complexa_2022	-989.53	0.0	-1351.2183	-627.8417	True
Boal_2024	Complexa_2023	-925.5267	0.0	-1287.2149	-563.8384	True
Boal_2024	Complexa_2024	-1158.1833	0.0	-1519.8716	-796.4951	True
Boal_2024	Malvasia_2022	-818.1133	0.0	-1179.8016	-456.4251	True
Boal_2024	Malvasia_2023	268.86	0.3917	-92.8283	630.5483	False
Boal_2024	Malvasia_2024	-506.67	0.0006	-868.3583	-144.9817	True
Boal_2024	Sercial_2022	386.9833	0.0249	25.2951	748.6716	True
Boal_2024	Sercial_2023	469.0933	0.002	107.4051	830.7816	True
Boal_2024	Sercial_2024	1077.36	0.0	715.6717	1439.0483	True
Boal_2024	Terrantez_2022	-208.1333	0.8083	-569.8216	153.5549	False
Boal_2024	Terrantez_2023	-551.31	0.0001	-912.9983	-189.6217	True
Boal_2024	Terrantez_2024	278.8767	0.3294	-82.8116	640.5649	False
Boal_2024	Tinta Negra_2022	-784.54	0.0	-1146.2283	-422.8517	True
Boal_2024	Tinta Negra_2023	-686.0267	0.0	-1047.7149	-324.3384	True
Boal_2024	Tinta Negra_2024	-973.65	0.0	-1335.3383	-611.9617	True
Boal_2024	Verdelho_2022	341.0167	0.0853	-20.6716	702.7049	False
Boal_2024	Verdelho_2023	1019.61	0.0	657.9217	1381.2983	True
Boal_2024	Verdelho_2024	1547.7367	0.0	1186.0484	1909.4249	True
Complexa_2022	Complexa_2023	64.0033	1.0	-297.6849	425.6916	False
Complexa_2022	Complexa_2024	-168.6533	0.9624	-530.3416	193.0349	False
Complexa_2022	Malvasia_2022	171.4167	0.9564	-190.2716	533.1049	False
Complexa_2022	Malvasia_2023	1258.39	0.0	896.7017	1620.0783	True
Complexa_2022	Malvasia_2024	482.86	0.0013	121.1717	844.5483	True

Complexa_2022	Sercial_2022	1376.5133	0.0	1014.8251	1738.2016	True
Complexa_2022	Sercial_2023	1458.6233	0.0	1096.9351	1820.3116	True
Complexa_2022	Sercial_2024	2066.89	0.0	1705.2017	2428.5783	True
Complexa_2022	Terrantez_2022	781.3967	0.0	419.7084	1143.0849	True
Complexa_2022	Terrantez_2023	438.22	0.0054	76.5317	799.9083	True
Complexa_2022	Terrantez_2024	1268.4067	0.0	906.7184	1630.0949	True
Complexa_2022	Tinta Negra_2022	204.99	0.826	-156.6983	566.6783	False
Complexa_2022	Tinta Negra_2023	303.5033	0.2033	-58.1849	665.1916	False
Complexa_2022	Tinta Negra_2024	15.88	1.0	-345.8083	377.5683	False
Complexa_2022	Verdelho_2022	1330.5467	0.0	968.8584	1692.2349	True
Complexa_2022	Verdelho_2023	2009.14	0.0	1647.4517	2370.8283	True
Complexa_2022	Verdelho_2024	2537.2667	0.0	2175.5784	2898.9549	True
Complexa_2023	Complexa_2024	-232.6567	0.6472	-594.3449	129.0316	False
Complexa_2023	Malvasia_2022	107.4133	0.9998	-254.2749	469.1016	False
Complexa_2023	Malvasia_2023	1194.3867	0.0	832.6984	1556.0749	True
Complexa_2023	Malvasia_2024	418.8567	0.0098	57.1684	780.5449	True
Complexa_2023	Sercial_2022	1312.51	0.0	950.8217	1674.1983	True
Complexa_2023	Sercial_2023	1394.62	0.0	1032.9317	1756.3083	True
Complexa_2023	Sercial_2024	2002.8867	0.0	1641.1984	2364.5749	True
Complexa_2023	Terrantez_2022	717.3933	0.0	355.7051	1079.0816	True
Complexa_2023	Terrantez_2023	374.2167	0.0356	12.5284	735.9049	True
Complexa_2023	Terrantez_2024	1204.4033	0.0	842.7151	1566.0916	True
Complexa_2023	Tinta Negra_2022	140.9867	0.9942	-220.7016	502.6749	False
Complexa_2023	Tinta Negra_2023	239.5	0.5979	-122.1883	601.1883	False
Complexa_2023	Tinta Negra_2024	-48.1233	1.0	-409.8116	313.5649	False
Complexa_2023	Verdelho_2022	1266.5433	0.0	904.8551	1628.2316	True
Complexa_2023	Verdelho_2023	1945.1367	0.0	1583.4484	2306.8249	True
Complexa_2023	Verdelho_2024	2473.2633	0.0	2111.5751	2834.9516	True
Complexa_2024	Malvasia_2022	340.07	0.0874	-21.6183	701.7583	False
Complexa_2024	Malvasia_2023	1427.0433	0.0	1065.3551	1788.7316	True
Complexa_2024	Malvasia_2024	651.5133	0.0	289.8251	1013.2016	True
Complexa_2024	Sercial_2022	1545.1667	0.0	1183.4784	1906.8549	True

Complexa_2024	Sercial_2023	1627.2767	0.0	1265.5884	1988.9649	True
Complexa_2024	Sercial_2024	2235.5433	0.0	1873.8551	2597.2316	True
Complexa_2024	Terrantez_2022	950.05	0.0	588.3617	1311.7383	True
Complexa_2024	Terrantez_2023	606.8733	0.0	245.1851	968.5616	True
Complexa_2024	Terrantez_2024	1437.06	0.0	1075.3717	1798.7483	True
Complexa_2024	Tinta Negra_2022	373.6433	0.0361	11.9551	735.3316	True
Complexa_2024	Tinta Negra_2023	472.1567	0.0018	110.4684	833.8449	True
Complexa_2024	Tinta Negra_2024	184.5333	0.9186	-177.1549	546.2216	False
Complexa_2024	Verdelho_2022	1499.2	0.0	1137.5117	1860.8883	True
Complexa_2024	Verdelho_2023	2177.7933	0.0	1816.1051	2539.4816	True
Complexa_2024	Verdelho_2024	2705.92	0.0	2344.2317	3067.6083	True
Malvasia_2022	Malvasia_2023	1086.9733	0.0	725.2851	1448.6616	True
Malvasia_2022	Malvasia_2024	311.4433	0.1713	-50.2449	673.1316	False
Malvasia_2022	Sercial_2022	1205.0967	0.0	843.4084	1566.7849	True
Malvasia_2022	Sercial_2023	1287.2067	0.0	925.5184	1648.8949	True
Malvasia_2022	Sercial_2024	1895.4733	0.0	1533.7851	2257.1616	True
Malvasia_2022	Terrantez_2022	609.98	0.0	248.2917	971.6683	True
Malvasia_2022	Terrantez_2023	266.8033	0.4052	-94.8849	628.4916	False
Malvasia_2022	Terrantez_2024	1096.99	0.0	735.3017	1458.6783	True
Malvasia_2022	Tinta Negra_2022	33.5733	1.0	-328.1149	395.2616	False
Malvasia_2022	Tinta Negra_2023	132.0867	0.9973	-229.6016	493.7749	False
Malvasia_2022	Tinta Negra_2024	-155.5367	0.983	-517.2249	206.1516	False
Malvasia_2022	Verdelho_2022	1159.13	0.0	797.4417	1520.8183	True
Malvasia_2022	Verdelho_2023	1837.7233	0.0	1476.0351	2199.4116	True
Malvasia_2022	Verdelho_2024	2365.85	0.0	2004.1617	2727.5383	True
Malvasia_2023	Malvasia_2024	-775.53	0.0	-1137.2183	-413.8417	True
Malvasia_2023	Sercial_2022	118.1233	0.9994	-243.5649	479.8116	False
Malvasia_2023	Sercial_2023	200.2333	0.8512	-161.4549	561.9216	False
Malvasia_2023	Sercial_2024	808.5	0.0	446.8117	1170.1883	True
Malvasia_2023	Terrantez_2022	-476.9933	0.0016	-838.6816	-115.3051	True
Malvasia_2023	Terrantez_2023	-820.17	0.0	-1181.8583	-458.4817	True
Malvasia_2023	Terrantez_2024	10.0167	1.0	-351.6716	371.7049	False

Malvasia_2023	Tinta Negra_2022	-1053.4	0.0	-1415.0883	-691.7117	True
Malvasia_2023	Tinta Negra_2023	-954.8867	0.0	-1316.5749	-593.1984	True
Malvasia_2023	Tinta Negra_2024	-1242.51	0.0	-1604.1983	-880.8217	True
Malvasia_2023	Verdelho_2022	72.1567	1.0	-289.5316	433.8449	False
Malvasia_2023	Verdelho_2023	750.75	0.0	389.0617	1112.4383	True
Malvasia_2023	Verdelho_2024	1278.8767	0.0	917.1884	1640.5649	True
Malvasia_2024	Sercial_2022	893.6533	0.0	531.9651	1255.3416	True
Malvasia_2024	Sercial_2023	975.7633	0.0	614.0751	1337.4516	True
Malvasia_2024	Sercial_2024	1584.03	0.0	1222.3417	1945.7183	True
Malvasia_2024	Terrantez_2022	298.5367	0.2254	-63.1516	660.2249	False
Malvasia_2024	Terrantez_2023	-44.64	1.0	-406.3283	317.0483	False
Malvasia_2024	Terrantez_2024	785.5467	0.0	423.8584	1147.2349	True
Malvasia_2024	Tinta Negra_2022	-277.87	0.3354	-639.5583	83.8183	False
Malvasia_2024	Tinta Negra_2023	-179.3567	0.9355	-541.0449	182.3316	False
Malvasia_2024	Tinta Negra_2024	-466.98	0.0022	-828.6683	-105.2917	True
Malvasia_2024	Verdelho_2022	847.6867	0.0	485.9984	1209.3749	True
Malvasia_2024	Verdelho_2023	1526.28	0.0	1164.5917	1887.9683	True
Malvasia_2024	Verdelho_2024	2054.4067	0.0	1692.7184	2416.0949	True
Sercial_2022	Sercial_2023	82.11	1.0	-279.5783	443.7983	False
Sercial_2022	Sercial_2024	690.3767	0.0	328.6884	1052.0649	True
Sercial_2022	Terrantez_2022	-595.1167	0.0	-956.8049	-233.4284	True
Sercial_2022	Terrantez_2023	-938.2933	0.0	-1299.9816	-576.6051	True
Sercial_2022	Terrantez_2024	-108.1067	0.9998	-469.7949	253.5816	False
Sercial_2022	Tinta Negra_2022	-1171.5233	0.0	-1533.2116	-809.8351	True
Sercial_2022	Tinta Negra_2023	-1073.01	0.0	-1434.6983	-711.3217	True
Sercial_2022	Tinta Negra_2024	-1360.6333	0.0	-1722.3216	-998.9451	True
Sercial_2022	Verdelho_2022	-45.9667	1.0	-407.6549	315.7216	False
Sercial_2022	Verdelho_2023	632.6267	0.0	270.9384	994.3149	True
Sercial_2022	Verdelho_2024	1160.7533	0.0	799.0651	1522.4416	True
Sercial_2023	Sercial_2024	608.2667	0.0	246.5784	969.9549	True
Sercial_2023	Terrantez_2022	-677.2267	0.0	-1038.9149	-315.5384	True
Sercial_2023	Terrantez_2023	-1020.4033	0.0	-1382.0916	-658.7151	True

Sercial_2023	Terrantez_2024	-190.2167	0.897	-551.9049	171.4716	False
Sercial_2023	Tinta Negra_2022	-1253.6333	0.0	-1615.3216	-891.9451	True
Sercial_2023	Tinta Negra_2023	-1155.12	0.0	-1516.8083	-793.4317	True
Sercial_2023	Tinta Negra_2024	-1442.7433	0.0	-1804.4316	-1081.0551	True
Sercial_2023	Verdelho_2022	-128.0767	0.9982	-489.7649	233.6116	False
Sercial_2023	Verdelho_2023	550.5167	0.0001	188.8284	912.2049	True
Sercial_2023	Verdelho_2024	1078.6433	0.0	716.9551	1440.3316	True
Sercial_2024	Terrantez_2022	-1285.4933	0.0	-1647.1816	-923.8051	True
Sercial_2024	Terrantez_2023	-1628.67	0.0	-1990.3583	-1266.9817	True
Sercial_2024	Terrantez_2024	-798.4833	0.0	-1160.1716	-436.7951	True
Sercial_2024	Tinta Negra_2022	-1861.9	0.0	-2223.5883	-1500.2117	True
Sercial_2024	Tinta Negra_2023	-1763.3867	0.0	-2125.0749	-1401.6984	True
Sercial_2024	Tinta Negra_2024	-2051.01	0.0	-2412.6983	-1689.3217	True
Sercial_2024	Verdelho_2022	-736.3433	0.0	-1098.0316	-374.6551	True
Sercial_2024	Verdelho_2023	-57.75	1.0	-419.4383	303.9383	False
Sercial_2024	Verdelho_2024	470.3767	0.002	108.6884	832.0649	True
Terrantez_2022	Terrantez_2023	-343.1767	0.0808	-704.8649	18.5116	False
Terrantez_2022	Terrantez_2024	487.01	0.0011	125.3217	848.6983	True
Terrantez_2022	Tinta Negra_2022	-576.4067	0.0001	-938.0949	-214.7184	True
Terrantez_2022	Tinta Negra_2023	-477.8933	0.0015	-839.5816	-116.2051	True
Terrantez_2022	Tinta Negra_2024	-765.5167	0.0	-1127.2049	-403.8284	True
Terrantez_2022	Verdelho_2022	549.15	0.0001	187.4617	910.8383	True
Terrantez_2022	Verdelho_2023	1227.7433	0.0	866.0551	1589.4316	True
Terrantez_2022	Verdelho_2024	1755.87	0.0	1394.1817	2117.5583	True
Terrantez_2023	Terrantez_2024	830.1867	0.0	468.4984	1191.8749	True
Terrantez_2023	Tinta Negra_2022	-233.23	0.6431	-594.9183	128.4583	False
Terrantez_2023	Tinta Negra_2023	-134.7167	0.9966	-496.4049	226.9716	False
Terrantez_2023	Tinta Negra_2024	-422.34	0.0088	-784.0283	-60.6517	True
Terrantez_2023	Verdelho_2022	892.3267	0.0	530.6384	1254.0149	True
Terrantez_2023	Verdelho_2023	1570.92	0.0	1209.2317	1932.6083	True
Terrantez_2023	Verdelho_2024	2099.0467	0.0	1737.3584	2460.7349	True
Terrantez_2024	Tinta Negra_2022	-1063.4167	0.0	-1425.1049	-701.7284	True

Terrantez_2024	Tinta Negra_2023	-964.9033	0.0	-1326.5916	-603.2151	True
Terrantez_2024	Tinta Negra_2024	-1252.5267	0.0	-1614.2149	-890.8384	True
Terrantez_2024	Verdelho_2022	62.14	1.0	-299.5483	423.8283	False
Terrantez_2024	Verdelho_2023	740.7333	0.0	379.0451	1102.4216	True
Terrantez_2024	Verdelho_2024	1268.86	0.0	907.1717	1630.5483	True
Tinta Negra_2022	Tinta Negra_2023	98.5133	1.0	-263.1749	460.2016	False
Tinta Negra_2022	Tinta Negra_2024	-189.11	0.9014	-550.7983	172.5783	False
Tinta Negra_2022	Verdelho_2022	1125.5567	0.0	763.8684	1487.2449	True
Tinta Negra_2022	Verdelho_2023	1804.15	0.0	1442.4617	2165.8383	True
Tinta Negra_2022	Verdelho_2024	2332.2767	0.0	1970.5884	2693.9649	True
Tinta Negra_2023	Tinta Negra_2024	-287.6233	0.2799	-649.3116	74.0649	False
Tinta Negra_2023	Verdelho_2022	1027.0433	0.0	665.3551	1388.7316	True
Tinta Negra_2023	Verdelho_2023	1705.6367	0.0	1343.9484	2067.3249	True
Tinta Negra_2023	Verdelho_2024	2233.7633	0.0	1872.0751	2595.4516	True
Tinta Negra_2024	Verdelho_2022	1314.6667	0.0	952.9784	1676.3549	True
Tinta Negra_2024	Verdelho_2023	1993.26	0.0	1631.5717	2354.9483	True
Tinta Negra_2024	Verdelho_2024	2521.3867	0.0	2159.6984	2883.0749	True
Verdelho_2022	Verdelho_2023	678.5933	0.0	316.9051	1040.2816	True
Verdelho_2022	Verdelho_2024	1206.72	0.0	845.0317	1568.4083	True
Verdelho_2023	Verdelho_2024	528.1267	0.0003	166.4384	889.8149	True
